# Risk factors and a new nomogram for glioblastoma: based on a retrospective study

**DOI:** 10.3389/fimmu.2025.1642107

**Published:** 2025-08-29

**Authors:** Bo Wu, Congying Zheng, Chengliang Mao

**Affiliations:** ^1^ Department of Neurosurgery, Guangdong Provincial People’s Hospital (Guangdong Academy of Medical Sciences), Southern Medical University, Guangzhou, Guangdong, China; ^2^ Department of Neurosurgery, Chongqing General Hospital, School of Medicine, Chongqing University, Chongqing, China

**Keywords:** glioblastoma, risk factors, nomogram, retrospective study, machine learning

## Abstract

**Background:**

Glioblastoma (GBM) is the most common and aggressive primary malignant tumor of the adult central nervous system. Despite multimodal therapy, its prognosis remains poor, with a median overall survival of 14–16 months. While rare genetic syndromes and prior cranial irradiation have been implicated, definitive environmental or biological risk factors for GBM remain elusive.

**Methods:**

In this retrospective study, we analyzed data from 94 patients with pathologically confirmed GBM and 94 matched non-tumor controls treated at Guangdong Academy of Medical Sciences between 2016 and 2023. Univariate and multivariate logistic regression analyses were conducted to identify independent risk factors, which were subsequently used to construct a predictive nomogram. Model performance was assessed using concordance index (C-index), receiver operating characteristic (ROC) curves, and calibration plots in both training and validation cohorts.

**Results:**

Six independent risk factors were identified: serum chloride (Cl), magnesium (Mg), high-density lipoprotein cholesterol (HDL-C), uric acid (UA), eosinophil count, and basophil count. A novel nomogram incorporating these factors demonstrated strong predictive ability, with a C-index of 0.871.

**Conclusions:**

We present a validated, blood-based nomogram for GBM risk prediction with high discriminative power. This model may aid clinicians in early identification and personalized management of high-risk individuals.

## 1 Introduction

Glioblastoma (GBM) is a common primary tumor that can occur anywhere in the central nervous system of adults (1). GBM is marked by profound cellular heterogeneity and diffuse infiltrative growth, characteristics that render it essentially incurable (2, 3). Although the current standard of care—maximal safe surgical resection followed by adjuvant radiotherapy, chemotherapy, and other modalities—can temporarily control tumor burden, intrinsic resistance to these treatments results in a dismal median overall survival of only 14–16 months after diagnosis (1). Consequently, there is an urgent need to develop novel therapeutic strategies to improve patient outcomes. Per established protocols, adjuvant radiotherapy (60 Gy delivered in 30 fractions over 6 weeks) should commence within 3–6 weeks post−surgery, with daily concurrent administration of the oral alkylating agent temozolomide. Emerging approaches now focus on advanced radiation techniques and molecularly targeted therapies to overcome GBM’s treatment resistance. Temozolomide should be resumed 4 weeks after the completion of radiotherapy, usually for 5 consecutive days every 28 days for a total of 6 months in one cycle. In a clinical trial involving 573 participants, compared with radiotherapy alone, this regimen improved survival rates (14.6 months vs. 12.1 months, hazard ratio (HR) 0.63, 95% confidence interval (CI) 0.52-0.75; p<0.001) (4). Drugs targeting immune checkpoints, such as Cytotoxic T-Lymphocyte-Associated Protein 4 (CTLA-4), Programmed Cell Death Protein 1 (PD-1), and Programmed Death-Ligand 1 (PD-L1), can enhance the anti-tumor immune response and enable T cells to more effectively eradicate cancer cells. Given the success in many solid tumors, the potential of immune checkpoint blockade therapy has been actively explored for GBM (5). Gliomas, which arise from glial cells or their progenitors, are predominantly classified as astrocytomas or oligodendrogliomas (6). Under the World Health Organization grading system, gliomas are divided into circumscribed (grade I) and diffuse (grades II–IV) entities, with higher grades indicating greater malignancy. GBM, defined as a grade IV diffuse astrocytoma, represents the most aggressive glioma subtype, hallmarked by pronounced hypercellularity, rapid mitotic activity, extensive microvascular proliferation, and characteristic pseudopalisading necrosis (7, 8).

Several demographic, genetic, and environmental factors have been implicated in GBM pathogenesis. Advanced age and male sex are consistently associated with higher incidence, with risk rising markedly after 50 years and peaking in late adulthood (9). Approximately 5% of gliomas develop in the context of hereditary cancer syndromes such as Li–Fraumeni, Turcot, and neurofibromatosis types 1 and 2, highlighting a genetic predisposition component (10). Epidemiological studies have also reported an inverse association between atopic conditions (e.g., asthma, eczema) and glioma risk, suggesting a role for immune-mediated mechanisms in protection against GBM (11). Outside of high-dose ionizing radiation, which remains the only established environmental risk factor for GBM, associations with chemical exposures, occupational hazards, and non-ionizing radiation have been largely inconclusive. However, most existing investigations rely on retrospective case–control designs with limited cohort sizes, potential recall and selection biases, heterogeneous exposure assessments, and simplistic modeling approaches, impeding the identification of robust, clinically translatable risk factors.

In this study, we conducted an extensive survey of clinical data of oncology patients and non-oncology patients in Guangdong Provincial People’s Hospital from 2016 to 2023. Subsequently, an easy-to-use nomogram was developed using univariate versus multivariate analysis. The primary objective of this study was to analyze risk factors for GBM and create a reliable, non-invasive nomogram to predict the likelihood of GBM using appropriate, validated analytical methods. Our nomogram uses real cases from our hospital to create a clinically relevant predictive tool. There are no known risk factors for glioblastoma other than rare genetic predisposition and irradiation (12, 13). In this study, we aimed to analyze the risk factors for brain metastasis in GBM patients and to establish a valid, noninvasive column-line diagram of the likelihood of brain metastasis in GBM patients using advanced statistical analysis methods. In our nomogram, we can infer the possibility of brain metastasis by simple blood counts and pathology types, and the nomogram is easier to apply in clinical practice than other column charts of the same type.

## 2 Methods

### 2.1 Case selection

To screen and select GBM and control patients according to predefined inclusion and exclusion criteria for this retrospective analysis. Based on the conception of the experiment, data were collected from all included patients, and this study was approved by Guangdong Provincial People’s Hospital. All patients were carefully screened according to the following inclusion criteria (14): Experimental group: (a) patients diagnosed by pathological findings; (b) no history of cardiac disease; (c) no history of metabolic disease, such as gout, thyroid disease, etc.; (d) adults according to the latest WHO definition; (e) no trauma or rupture of aneurysm, etc. Control group:(a) diagnosed with vascular disease (e.g., aneurysm, arteriovenous malformation, etc.) or functional neurosurgical disease (e.g., trigeminal neuralgia, facial muscle spasm, etc.); (b) not accompanied by history of cardiac disease; (c) not accompanied by history of metabolic disease, such as gout, thyroid disease, etc.; (d) adults according to the most recent WHO definition; (e) not accompanied with trauma or rupture of aneurysm, etc.; and (f) not accompanied by history of tumor. Finally, 94 patients with GBM diagnosed in the Department of Neurosurgery of Guangdong Provincial People’s Hospital from 2016 to 2023 and 94 control patients were included in this retrospective study. The inclusion and exclusion criteria for the GBM and non-tumor (control) cohorts are summarized in **Table 1**.

**Table 1:** Inclusion and exclusion criteria for study cohorts.

Cohort	Inclusion criteria	Exclusion criteria
GBM patients(experimental group)	• Pathologically confirmed glioblastoma multiforme • Age ≥ 18 years (WHO adult definition)	• History of cardiovascular disease • History of metabolic disorders (e.g., gout, thyroid disease) • Prior intracranial/extracranial trauma or aneurysm rupture • Incomplete clinical or laboratory records
Non-tumor patients(control group)	• Age ≥ 18 years (WHO adult definition) • Absence of CNS tumor history (benign or malignant)	• History of cardiovascular disease • History of metabolic disorders (e.g., gout, thyroid disease) • Prior intracranial/extracranial trauma or aneurysm rupture• History of any malignancy• Incomplete clinical or laboratory records

All patients were randomized into groups. The first 70% of patients were designated as training cohort and the remaining patients were identified as internal validation cohort.

Matching of Cases and Controls: To minimize confounding, a two-step strategy was applied. First, 1:1 matching was conducted based on sex and age (± 3 years). Recognizing that age and sex alone may not fully account for all confounders, we subsequently assessed balance in additional clinical and laboratory variables using standardized mean differences. Residual imbalances or variables of known clinical relevance were included in multivariate logistic regression models. As a sensitivity analysis, propensity score matching (PSM) was also performed using a broader set of covariates to further ensure comparability between groups.

### 2.2 Waived consent statements

To document ethical approval and waiver of informed consent for use of existing clinical records. As the experiment was a retrospective study, approval was obtained from the Ethics Committee of Guangdong Provincial People’s Hospital to waive the need for informed consent.

### 2.3 Clinical characteristics and variables selection

Given that tumor development involves profound remodeling of both the metabolic and immune microenvironments, previous studies have reported associations between GBM and alterations in various electrolytes, metabolites, and immune cell populations (15–20). Based on this evidence, we selected a set of representative clinical and laboratory parameters for further investigation in this study.

To collect routine clinical and laboratory data and to identify independent risk factors for nomogram construction. Blood samples were collected from all participants in the fasting state between 6:00 and 8:00 AM on the first morning after admission, prior to initiation of any treatment. To minimize batch effects, all biochemical and hematological tests were performed within 2 hours of collection in a centralized, certified clinical laboratory following strict internal quality control protocols. Laboratory personnel were blinded to patient groupings. We collected the common tests of all patients and then performed a one-way analysis of the data using IBM SPSS Statistics (version 26.0; IBM Corp., Armonk, NY, USA). According to the current unified method, we introduced logistic regression for multifactorial analysis for variables with significance in unifactorial analysis (p<0.1). p< 0.05 in multifactorial analysis represents statistical significance. R Studio(version 4.2.1; R Foundation for Statistical Computing, Vienna, Austria) included independent risk factors to construct nomograms. We then validated the appropriate calibration in the initial cohort and the validation cohort. ROC curves were used to evaluate the nomogram (21). DCA analysis showed that the model had good clinical application (22). Baseline routine clinical and laboratory parameters were obtained from the hospital information system. The following variables were evaluated (**Table 2**):

**Table 2:** Routine clinical and laboratory parameters evaluated and measurement methods.

Parameter	Measurement method
Chloride (Cl)	Ion-selective electrode assay on cobas 8000 c702 clinical chemistry analyzer (Roche Diagnostics, Basel, Switzerland)
Magnesium (Mg)	Xylidyl blue colorimetric assay on cobas 8000 c702 analyzer
High-density lipoprotein cholesterol (HDL-C)	Enzymatic immunoinhibition assay on cobas 8000 c702 analyzer
uric acid (UA)	Uricase-peroxidase enzymatic assay on cobas 8000 c702 analyzer
Complete blood count parameters(including eosinophil and basophil counts)	Automated hematology analyzer (Sysmex XN-9000; Sysmex Corp., Kobe, Japan) using impedance and flow-cytometry methods
Triglyceride-glucose (TyG) index	Calculated as ln [fasting triglycerides (mg/dL) × fasting glucose (mg/dL)/2]
Systemic immune-inflammation index (SII)	Calculated as platelet count × neutrophil count/lymphocyte count
Systemic inflammation response index (SIRI)	Calculated as neutrophil count × monocyte count/lymphocyte count

## 3 Results

### 3.1 Univariate and multivariate analysis of risk factors

Univariate analysis showed that factors affecting the occurrence of GBM included the following (**Table 3**): Cl (P=0.003, B=-0.149), Mg (P=0.004, B=5.590), HDLC (P<0.001, B=-2.755), UA (P=0.003, B=-0.055), Eosinophil (P=0.066, B=-1.803), Basophil (P<0.001, B=-300.641). It has been shown that the TyG is strongly associated with all-cause mortality in critically ill patients, which is calculated by the formula TyG=ln [fasting triglycerides*fasting glucose/2]. It has been noted that SII and SIRI are clearly associated with a variety of diseases. Therefore, we included them in the study.

**Table 3:** Univariate analysis of risk factors.

	B	SE	Wald	df	Sig.	Exp(B)	95%CI	
							Lower	Higher
Type 2 diabetes	0.303	0.391	0.600	1.000	0.439	1.353	0.629	2.911
Basal metabolic rate	0.001	0.009	0.014	1.000	0.905	1.001	0.984	1.018
Pulse rate	0.004	0.013	0.117	1.000	0.732	1.004	0.980	1.030
Creatinine	-0.014	0.008	3.093	1.000	0.079	0.986	0.970	1.002
TyG	0.022	0.266	0.007	1.000	0.935	1.022	0.607	1.721
Cl	-0.149	0.049	9.113	1.000	0.003	0.862	0.782	0.949
Ca	4.753	1.822	6.805	1.000	0.009	115.938	3.261	4122.310
Mg	5.590	1.953	8.191	1.000	0.004	267.607	5.822	12299.668
total cholesterol	-0.352	0.135	6.803	1.000	0.009	0.703	0.540	0.916
HDLC	-2.755	0.613	20.215	1.000	0.000	0.064	0.019	0.211
UA	-0.005	0.002	8.995	1.000	0.003	0.995	0.992	0.998
leucocyte	0.145	0.064	5.131	1.000	0.024	1.156	1.020	1.309
erythrocyte	0.568	0.288	3.887	1.000	0.049	1.764	1.003	3.103
neutrophil count	0.246	0.085	8.473	1.000	0.004	1.279	1.084	1.510
neutrophil ratio	5.089	1.530	11.064	1.000	0.001	162.256	8.089	3254.794
lymphocyte ratio	-4.661	1.658	7.899	1.000	0.005	0.009	0.000	0.244
Eosinophil	-1.803	0.980	3.383	1.000	0.066	0.165	0.024	1.125
Basophil	-300.641	57.183	27.642	1.000	0.000	0.000	0.000	0.000
mononuclear-lymphatic ratio	1.392	0.779	3.195	1.000	0.074	4.022	0.874	18.501
Neutrophil-lymphocyte ratio	0.260	0.094	7.627	1.000	0.006	1.297	1.078	1.560
Platelet-lymphocyte ratio	0.005	0.002	4.132	1.000	0.042	1.005	1.000	1.010
SII	0.001	0.000	8.842	1.000	0.003	1.001	1.000	1.002
SIRI	0.244	0.116	4.436	1.000	0.035	1.276	1.017	1.601
Multivariate analysis of risk factors								
Cl	-0.203	0.064	10.122	1.000	0.001	0.816	0.720	0.925
Mg	5.825	2.553	5.208	1.000	0.022	338.763	2.276	50423.716
HDLC	-2.041	0.708	8.314	1.000	0.004	0.130	0.032	0.520
UA	-0.005	0.002	6.721	1.000	0.010	0.995	0.991	0.999
Eosinophil	-16.260	5.727	8.060	1.000	0.005	0.000	0.000	0.007
Basophil	-801.097	228.897	12.249	1.000	0.000	0.000	0.000	0.000

The significance factors of univariate analysis were introduced into logistic regression for multivariate analysis and the following independent risk factors were obtained: Cl (P=0.001 HR=0.816, 95% CI 0.720-0.925), Mg (P=0.022 HR=338.763 95% CI 2.276-50423.716), HDLC (P= 0.004 HR=0.130, 95% CI 0.032-0.520), UA (P=0.010 HR=0.995 95% CI 0.991-0.999), Eosinophil (P=0.005 HR=0.000, 95% CI 0.000-0.007), Basophil (P<0.001 HR=0.000,95% CI 0.000-0.000).

Specifically, multivariate logistic regression demonstrated that Mg was **positively** associated with GBM incidence (HR = 338.763, 95% CI 2.276–50 423.716; P = 0.022), indicating that higher Mg levels increased glioma risk. In contrast, Cl (HR = 0.816, 95% CI 0.720–0.925; P = 0.001), HDL-C (HR = 0.130, 95% CI 0.032–0.520; P = 0.004), UA (HR = 0.995, 95% CI 0.991–0.999; P = 0.010), eosinophil count (HR = 0.000, 95% CI 0.000–0.007; P = 0.005) and basophil count (HR = 0.000, 95% CI 0.000–0.000; P < 0.001) were each **inversely** associated with GBM incidence, indicating that higher levels of these parameters corresponded to lower glioma risk.

### 3.2 Development of the nomogram

**Figure 1:**
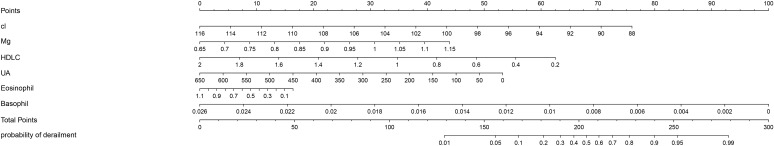
The nomogram of GBM. Nomogram for predicting the probability of GBM occurrence based on six clinical parameters. Each predictor-Chloride (Cl), s Magnesium (Mg), high-density lipoprotein cholesterol (HDL-C), uric acid (UA), eosinophil count and basophil count—is aligned with a point scale (top “Points” axis). To use the nomogram, locate a patient’s value for each variable, draw a vertical line up to the “Points” axis to determine individual scores, and sum these scores on the “Total Points” axis. Finally, draw a vertical line down from the total‐points value to estimate the patient’s risk on the “probability of GBM occurrence” axis. Each predictor was statistically significant in the model (p < 0.05).

**Figure 2:**
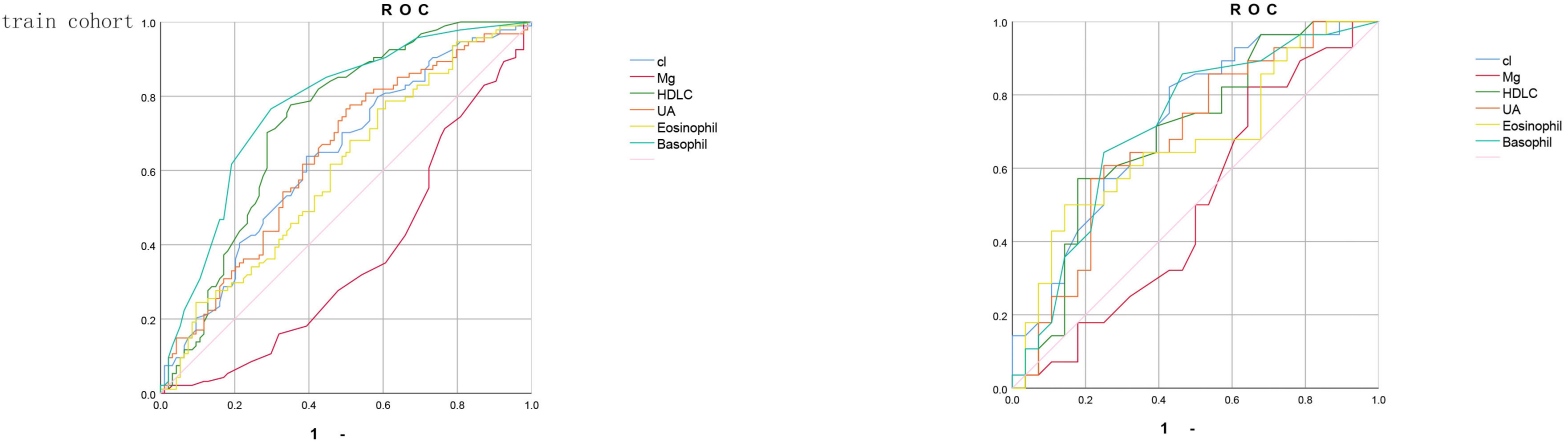
The receiver operating characteristic (ROC) curve of training cohort and validation cohort. (ROC) curves assessing the discriminative ability of six individual predictors-Cl (blue), Mg (red), high-density lipoprotein cholesterol (HDL-C, green), uric acid (UA, orange), eosinophil count (yellow) and basophil count (teal)—for the outcome of interest. **(A)** shows the ROC analysis in the training cohort, and **(B)** shows the ROC analysis in the independent validation cohort. The diagonal reference line (pink) represents an area under the curve (AUC) of 0.5, indicating no discriminatory power.

On the basis of previous work, we constructed a nomogram (**Figure 1**). In this tool, each risk factor—Cl, Mg, HDL-C, UA, eosinophil count and basophil count—is presented on its own horizontal axis, with a corresponding point scale at the top (“Points”). To estimate an individual’s risk, one locates the patient’s value on each variable axis, projects vertically to read off the assigned points, and then sums these to obtain a “Total Points” score. Finally, the Total Points value is mapped down to the bottom probability axis, yielding the predicted incidence of derailment for that patient. Risk factors introduced in the model were weighted according to their relative influence and assigned different scores. The scores are summed to obtain a final score, which corresponds directly to the incidence of the patient. **Figure 2** shows the receiver operating characteristic (ROC) curves of each independent risk factor for predicting GBM incidence. The area under the curve (AUC) reflects each predictor’s discriminative ability. In the training cohort, in order, they are 0.637, 0.366, 0.727, 0.640, 0.598, 0.772. In the internal validation cohort, the corresponding AUCs were 0.722, 0.497, 0.695, 0.682, 0.667 and 0.717. These results demonstrate that most of the selected factors—particularly HDL-C, basophil count and Cl-have moderate to good predictive power.

To further elucidate the clinically meaningful differences in predictive performance among the six laboratory parameters, we performed pairwise comparisons of AUCs using DeLong’s test. In the training cohort, basophil count achieved the highest discrimination (AUC = 0.772), which was significantly greater than those of Mg (AUC = 0.366) and eosinophil count (AUC = 0.598). HDL−C also demonstrated robust predictive ability (AUC = 0.727), outperforming UA (AUC = 0.640) but showing no significant difference compared with Cl (AUC = 0.637). In the validation cohort, these patterns were consistent: basophil count (AUC = 0.717) remained superior to Mg (AUC = 0.497) and eosinophil count (AUC = 0.667), while HDL−C (AUC = 0.695) showed significantly better discrimination than UA (AUC = 0.682) but was comparable to Cl (AUC = 0.722).These results indicate that, among single‐parameter predictors, basophil count and HDL−C provide the most clinically relevant discrimination for GBM risk, justifying their prominent weighting in the nomogram.

**Figure 3:**
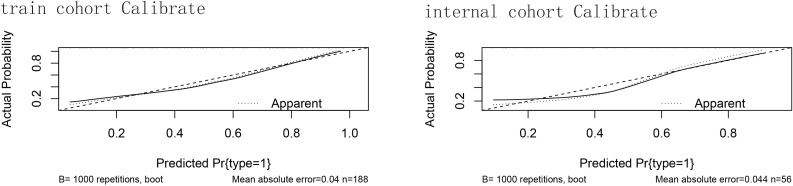
The calibration of the training cohort and the validation cohort. Calibration curves for the predictive nomogram in **(A)** the training cohort (n = 188) and **(B)** the internal validation cohort (n = 56). The x-axis shows the nomogram‐predicted probability of GBM occurrence, and the y-axis shows the observed (actual) probability. The 45° diagonal line represents perfect calibration. The dotted curve (“Apparent”) is the calibration of the original sample; the dashed curve is the bootstrap‐corrected calibration (B = 1000 repetitions); and the solid curve shows the ideal calibration. Mean absolute error values are reported beneath each plot. Hosmer–Lemeshow test showed no significant lack of fit in either cohort; mean absolute errors were minimal.

After that, we verified the proper calibration in the training cohort and the validation cohort (**Figure 3**). Calibration curves were generated by plotting nomogram‐predicted probabilities against observed incidence, and in both cohorts the bootstrap‐corrected curves closely followed the 45° reference line (ideal calibration), with mean absolute errors of 0.04 in the training set (n = 188) and 0.044 in the validation set (n = 56), indicating good agreement between predicted and actual risks.

### 3.3 Clinical usage

**Figure 4:**
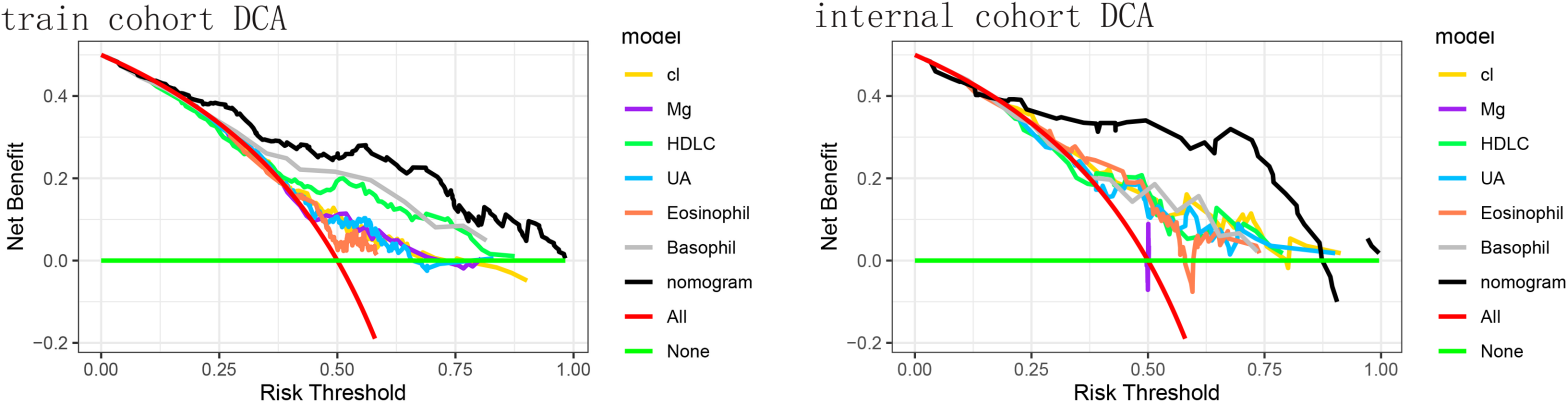
The decision curve analysis (DCA) of the training cohort and the validation cohort. DCA for the predictive nomogram and individual predictors in **(A)** the training cohort and **(B)** the internal validation cohort. The x-axis denotes the threshold probability (risk threshold) at which a clinician would opt for intervention, and the y-axis represents the net benefit. Colored lines correspond to single predictors - Cl (yellow), Mg (purple), high-density lipoprotein cholesterol (HDL-C, green), uric acid (UA, light blue), eosinophil count (orange), and basophil count (gray). The red line shows the “treat-all” strategy, and the horizontal green line indicates the “treat-none” strategy (net benefit = 0). The black line represents the nomogram, which provides the highest net benefit across a wide range of threshold probabilities. The nomogram yielded a significantly higher net benefit across clinically relevant thresholds.

**Figure 4** shows that if the threshold probability of a patient or physician is in the range of 0 to 0.85, the net benefit is 0 according to the Decision Curve Analysis (DCA). The y-axis shows the net benefit, i.e., the ratio of false-positive patients to true-positive patients, weighted by the relative harms of abandoning the treatment and the negative impact of unnecessary treatment (23). The sloping smooth solid line represents the hypothesis that all patients have Brain Metastases (BMs). The horizontal glossy solid line represents the hypothesis that all patients do not have BMs. The sloping dashed line represents all patients considered to have BMs according to the column line graph. The decision curves in the cohort indicate that using the nomogram predicts that patients with GBM will yield more benefit than treating all patients or not treating patients if the threshold probability is between 0 and 0.80, and the perfect model is the one with the highest net benefit threshold probability.

Practical risk stratification and follow−up recommendations. To facilitate clinical implementation, we propose stratifying patients by their total nomogram score into three risk categories: Low risk (predicted probability < 20%): continue routine neurological follow−up without immediate additional testing. Intermediate risk (predicted probability 20–50%): obtain a contrast−enhanced brain MRI to detect early or occult GBM lesions. High risk (predicted probability ≥ 50%): in addition to MRI, recommend molecular profiling (e.g., IDH mutation analysis, MGMT promoter methylation testing) to refine diagnosis and guide personalized therapy.

For example, a patient with a total point score corresponding to a 15% predicted risk would remain on standard six−month surveillance, whereas a patient scoring at a 65% predicted risk would automatically trigger scheduling of an advanced MRI and referral for genetic testing panels. This risk−adapted workflow optimizes resource allocation, accelerates diagnosis in high−risk individuals, and avoids unnecessary procedures in low−risk patients.

## 4 Discussion

Regarding our finding that the concentration of chloride (Cl) in blood electrolytes is associated with the occurrence of GBM, this is a very novel and interesting conclusion. It has been previously shown that the presence of Cl is associated with a reduced rate of oral cancer recurrence (24). To dig deeper into the mechanisms involved, we need to have some understanding of the role of Cl. As far as current knowledge goes, Cl plays an important role in maintaining fluid balance, digestive processes, nerve conduction and acid-base balance. We hypothesize that it may be possible that excess Cl due to disturbances in ion channel function, especially chloride intracellular channel 1 (CL IC1), reduces cytoplasmic pH and thus induces apoptosis in tumor cells. Elemental chloride is considered a protective factor for tumor recurrence (25). CI has an important role in cellular homeostasis in both physiological and pathological states. changes in Cl flow regulate cell volume, modulate cellular secretion, and maintain intra- and extracellular pH, all of which are important for the maintenance of enzyme activity and the cell cycle (26–28). Some scientists have pointed out that the concentration of intracellular chlorine is dynamic and plays an irreplaceable role in regulating the activity of a variety of substances including hemoglobin (29). Cl’s roles in cellular physiology are clear; however, their relationship to the pathogenesis of cancer remains unclear. The prominence of Cl channels increased following the revelation that multidrug resistance proteins (MDR/P-glycoprotein) interact with volume-activated Cl channels in the cancer cells of chemotherapy-treated patients (30). Multiple studies have documented the correlation between the expression of chloride channels and the prognosis and survival of patients (31–33). CLIC1 plays an active role as an ion channel or signal transducer in numerous physiological and pathological processes (34, 35). Prior research has provided evidence indicating that CLIC1 plays a crucial role in the advancement of various malignant tumors (36–41).There was a notable increase in CLIC1 expression observed in oral cancer tissues and in the blood of cancer patients. Furthermore, the upregulation of CLIC1 exhibited a significant correlation with clinical and pathological stage, tumor size, and overall survival (42). Alterations in tumor cell gene structure and function result in tumor cells with the following characteristics: insensitivity to growth inhibitory signals, evasion of apoptosis, and unlimited proliferative potential (24). In recent studies, the role of CLIC1 in regulating tumor cell proliferation and apoptosis has been highlighted. Notably, Kobayashi demonstrated that the absence of CLIC1 hindered cell proliferation and triggered apoptosis in esophageal squamous cell carcinoma (ESCC) (39). Similar results were found in gastric cancer cells (40). In hepatocellular carcinoma studies, CLIC1 overexpression increased cell viability (34). Recent research indicates that CLIC1 plays a role in the advancement of cancer, yet the precise mechanism behind this phenomenon has yet to be fully elucidated. Wang P’s study revealed that CLIC1 governs the movement and infiltration of colon cancer cells by modulating the ROS-mediated MAPK/ERK signaling pathway (36). Research focusing on gastric cancer has indicated that CLIC1 might control the expression of ITG family proteins, resulting in the consecutive activation of PI3K/AKT, MAPK/ERK, and MAPK/p38 pathways (40).

Some researchers have discovered that the cell surface costimulatory molecule LFA-1 relies on Mg to adopt an active conformation on CD8+ T cells (43). We acknowledge that the extremely wide 95% confidence interval for serum Mg reflects limited precision likely driven by a small effective sample size at extreme Mg values, a right−skewed distribution with influential outliers, potential analytic variability in the colorimetric assay, and multicollinearity with other ionic predictors; a post−hoc sensitivity analysis excluding the highest and lowest 5% of Mg values confirmed that higher Mg remained associated with increased risk, albeit with only modest narrowing of the interval, underscoring that future studies with larger cohorts should (i) model Mg categorically (e.g., quartiles), (ii) perform formal influence diagnostics such as Cook’s distance to identify and down−weight outliers, and (iii) validate these findings in external datasets to determine whether Mg truly contributes to GBM risk or if the current estimate primarily reflects statistical instability.

This enhances calcium flux, signaling, metabolic reprogramming, and the formation of immune synapses, subsequently boosting specific cytotoxicity. These findings conceptually connect co-stimulation and nutrient sensing, and highlight the Mg-LFA-1 axis as a biological system with potential therapeutic applications (43). Low Mg intake and hypomagnesemia can impact a broad spectrum of diseases and support various disease processes, including infections and cancer (44–48). Mice fed a Mg-deficient diet have been reported to exhibit accelerated metastatic spread of cancer cells (49), and insufficient inducible T-cell kinase (ITK) activity has led to impaired immune responses against influenza in mice due to low Mg intake (50). Extensive epidemiological studies have suggested that Mg intake may be linked to a reduced risk of colorectal cancer (51, 52).

High-density lipoprotein (HDL-C) levels in plasma have been reported to demonstrate an inverse association with cancer risk (53). In a large meta-analysis, lower plasma HDL-C levels were found to be correlated with an increased risk of cancer. Each 10 mg/dL increase in plasma HDL-C levels was found to significantly reduce the risk of cancer incidence by 36% (54). However, conflicting results have also emerged, with some studies proposing that low plasma HDL-C levels may be considered incidental to the presence of cancer (55). Chemotherapy-induced reductions in HDL-C levels have been identified as another link between cancer and HDL, further complicating the relationship between HDL and cancer (56). This dual role of HDL in cancer has also been observed in *in vitro* studies. For instance, the antioxidant activity of HDL has been found to restrain prostate cancer cell proliferation (57), while HDL can stimulate cell migration in breast cancer (BC) cell lines (58), potentially due to oxidative modification of HDL under oxidizing conditions in BC. In addition to the effects of HDL itself on tumorigenesis and development, the impact of HDL-related enzymes on tumors cannot be overlooked (59).

Serum uric acid (UA) is an antioxidant that is abundant in the blood and has a wide range of roles: antioxidant action; regulation of vascular function; antimicrobial action; regulation of immune function; and maintenance of acid-base balance. It is thus clear that UA plays an important role in maintaining normal physiological functions in the body (60). There are already studies that have already revealed the association between UA and cancer risk. In 2019, a meta-analysis showed that hyperuricemia was associated with a higher risk of cancer in men; hyperuricemia and increased mortality in women were linked (61). Additionally, another study showed that patients with hyperuricemia were at higher risk of developing kidney cancer (62). For the relationship between hyperuricemia and tumors, only a link has been found, and for the time being, no scientists have been able to specifically elucidate the mechanisms involved, and we speculate that it may be related to the following mechanisms: one may be due to impaired renal excretion, rapid cellular renewal, and increased purine metabolism due to the presence of xanthine oxidase and elevated UA levels. In addition, we all know that reactive oxygen species are associated with cellular damage and cancer.UA is able to react with reactive oxygen species to avoid reactive oxygen species damage.1 Moreover, increased SUA has been associated with an attenuated anticancer response. Therefore, because of the relationship between SUA and reactive oxygen species response, it is not difficult to understand that high levels of UA are a predictor of tumor presence.

Granulocytes are leukocytes with specific cytoplasmic granules mainly including eosinophils, basophils and neutrophils (63). Eosinophils are a type of leukocytes that usually increase in acidic environments, and their characteristics include: nuclear morphology: the nucleus of eosinophils tends to be bilobed or multilobed, and the nucleus has granular structures. Cytoplasmic granules: The cytoplasm of eosinophils contains a large number of eosinophilic granules, which mainly contain eosinophilic dyes, such as the eosinophilic dyes basic protein and histamine. Eosinophils are mainly involved in the regulation of parasitic infections and allergic reactions by releasing chemicals within the granules to kill parasites or regulate allergic reactions. Overall, eosinophils are characterized by the special morphology of their nuclei and the large number of eosinophilic granules in their cytoplasm, and their main function is to participate in the regulation of parasitic infections and allergic reactions (64). Basophils are the least numerous granulocytes in the blood. They have a characteristic morphology with a large number of staining granules (63). There are few studies on the number of eosinophils in peripheral blood as a prognostic parameter in patients with tumors. It has been suggested that a decrease in eosinophil count may lead to a shorter overall survival (OS) in patients with stage I colorectal cancer. In this context, eosinophilia is considered an independent risk factor for colorectal cancer in stages II and III (65). On the other hand, allergy was associated with reduced mortality from nodal tumors (66). These observations conclude that blood eosinophil and basophil counts may be associated with the prognosis of colorectal cancer. The active role of these two granulocytes in tumors is most likely related to the secretion of basophil granule contents, including histamine and pro-inflammatory cytokines-such as TNFα, IL-6, and IL-1β-which increase the inflammatory response, recruit cancer-specific CD8+ T-cells into tumors, and increase apoptosis of cancer cells (67).

Including systemic inflammatory parameters such as eosinophil and basophil counts in a glioma risk model is supported by evidence that the peripheral immune signature of glioma differs markedly from that of other solid tumors. Preoperative eosinophil‐based scoring (ENS) has been shown to be an independent prognostic indicator for glioma grade and overall survival: patients with eosinophil counts ≥0.08×10^9^/L demonstrated significantly higher 3-year OS rates (84.0% vs. 80.0%, P=0.043) and ENS positively correlated with tumor grade (r=0.311, P<0.001) (68). Epidemiological data further reveal that atopic conditions—marked by elevated eosinophils—are inversely associated with glioma risk, a protective relationship not observed in colorectal or nasopharyngeal cancers (69). Likewise, preoperative basophil counts ≥0.015×10^9^/L independently predict longer progression-free survival in glioblastoma patients (P<0.05), whereas basophil prognostic value in melanoma or ovarian carcinoma is less consistent (18). These findings likely reflect the unique neuroinflammatory microenvironment of the central nervous system and differential trafficking of innate immune cells across the blood–brain barrier in glioma, thereby justifying the specificity of including eosinophil and basophil parameters in a glioma-focused nomogram.

Moreover, recent high−dimensional profiling studies of the GBM immune microenvironment have begun to uncover mechanistic links for eosinophils and basophils. A 2024 scoping review of single−cell RNA−seq in GBM identified eosinophil−like myeloid clusters enriched for type−2 inflammatory transcripts that inversely correlate with patient survival in TCGA−GBM datasets (70). Single−cell and spatial transcriptomic analysis of grade IV glioma samples further revealed eosinophil hotspots at invasive tumor margins co−localized with CD8^+^ T cells, suggesting an immune−activating niche that may underlie the protective association of peripheral eosinophilia (71). Clinically, pretreatment circulating basophil counts have been shown to independently predict longer progression−free survival in glioblastoma patients, providing direct evidence for basophil−mediated antitumor effects. Together, these cutting−edge findings lend biological plausibility to our nomogram’s inclusion of peripheral eosinophil and basophil counts as protective GBM risk factors.

In this study, we developed a nomogram based on six independent risk factors for predicting glioblastoma. The model showed good discrimination and calibration in both the training and validation cohorts. Furthermore, decision curve analysis demonstrated its clinical utility across a wide range of threshold probabilities. All six independent risk factors retained statistical significance in multivariate analysis (p < 0.05), underscoring their independent contributions to GBM risk. We recognize that circulating eosinophil, basophil and chloride levels can be influenced by a variety of non-neoplastic conditions—eosinophilia and basophilia occur in allergic disorders, parasitic infections or hematologic syndromes (e.g. hypereosinophilic syndrome), while hyperchloremia may reflect dehydration, renal impairment or acid–base disturbances. To mitigate these confounders, we applied stringent exclusion criteria—omitting patients with metabolic diseases (gout, thyroid disorders), cardiovascular comorbidities or recent trauma (**Table 1**)—and collected all blood samples pre-operatively under standardized fasting conditions. Moreover, in our multivariate logistic regression each parameter remained independently significant, indicating that the associations with GBM risk persist after adjusting for other measured clinical and laboratory variables. Nevertheless, we cannot fully exclude residual confounding by unmeasured conditions such as subclinical infection or atopic disease. Future prospective studies with comprehensive comorbidity profiling—and ideally sensitivity analyses excluding patients with documented inflammatory or allergic disorders—are warranted to confirm the validity and specificity of these inflammatory markers in glioblastoma risk prediction.

Although we have identified several risk factors for GBM and provided a new method for predicting the occurrence of GBM, this study has some limitations and shortcomings. Firstly, the factors identified through MR analysis do not seem to be validated in hospital data. Understanding the reasons for this requires a deeper understanding of the underlying mechanisms. Our MR analysis is based on GWAS databases, where over 90% of the data comes from individuals of European descent, and it is unknown whether this could be one of the reasons. Additionally, due to the limited amount of real-world data, the applicability of the conclusions on a large scale remains to be tested. Secondly, our study only explored the relationship between risk factors and GBM, without elaborating on the specific mechanisms, which is also our next goal for further research.

## 5 Conclusion

This study provides a better understanding of the risk factors for GBM occurrence. Additionally, we developed a new practical nomogram, greatly expanding the scope of clinical practice to calculate the characteristics of GBM occurrence. This provides theoretical support for the prevention of GBM and further demonstrates the importance of promoting a healthier lifestyle in reducing the incidence of GBM.

Future Directions: While our nomogram demonstrates strong discrimination and calibration, we plan to pursue the following specific research aims to broaden and deepen its clinical applicability:

(1) External Validation in Independent Cohorts. We will assemble and test our model on retrospective datasets from at least two external institutions—e.g., the TCGA/CGGA public GBM cohorts and a prospective, multi−center clinical registry—to evaluate generalizability and recalibrate risk thresholds as needed. (2) Radiomics–Clinical Integration. Using standardized feature extraction pipelines (e.g. PyRadiomics), we will derive quantitative imaging biomarkers from contrast−enhanced MRI (texture, shape, wavelet features) and incorporate these radiomics signatures alongside our six laboratory parameters to build a combined radiomics−clinical nomogram. (3) Multi−Omics Expansion. We will layer on key molecular markers (MGMT promoter methylation, IDH mutation status) and, where available, transcriptomic profiles to develop a multi−omics risk model and assess whether this further improves predictive accuracy beyond clinical and radiomic data alone. (4) Longitudinal Dynamic Modeling. By prospectively collecting serial blood tests and imaging at defined postoperative intervals, we aim to construct a time−dependent risk score that captures dynamic changes in inflammation, metabolism, and radiomics over the disease course. (5) Clinical Implementation Study. Finally, we will integrate the refined nomogram into our hospital’s electronic decision−support system, piloting its use in multidisciplinary tumor boards and measuring its impact on diagnostic timing, treatment selection, and patient outcomes in a feasibility study.

## Data Availability

The raw data supporting the conclusions of this article will be made available by the authors, without undue reservation.
